# Seroprevalence of Human Immunodeficiency Virus (HIV), Hepatitis B Virus (HBV), and Hepatitis C Virus (HCV) Among Blood Donors in Borgou, Benin in 2023: A Cross-Sectional Study

**DOI:** 10.3390/v17081107

**Published:** 2025-08-12

**Authors:** Kamel-Dine Djaliri, Brice Boris Legba, Victorien Dougnon, Abdelsalam Tidjani, Lamine Baba-Moussa

**Affiliations:** 1 Research Unit in Applied Microbiology and Pharmacology of Natural Substances, Laboratory of Applied Microbiology, Polytechnic School of Abomey-Calavi, Cotonou 01 P.O. Box 2009, Benin; kameldine2005@yahoo.fr (K.-D.D.); legbaboris5@gmail.com (B.B.L.); 2 Laboratory for Research in Food Science and Nutrition, University of N’Djamena, N’Djamena P.O. Box 1117, Chad; abdelti@gmail.com; 3 Laboratory of Biology and Molecular Typing in Microbiology, Faculty of Sciences and Technology, University of Abomey-Calavi, Cotonou 01 P.O. Box 2009, Benin; laminesaid@yahoo.fr

**Keywords:** seroprevalence, blood donors, HIV, HBV, HCV, Borgou, Benin

## Abstract

Blood transfusion remains vital in healthcare but poses risks, particularly from transfusion-transmissible viral infections (TTVIs). This study aims to determine the seroprevalence of HIV, HBV, and HCV among blood donors in Borgou (Benin) in 2023. This prospective, cross-sectional study involved voluntary, non-remunerated blood donors recruited via mobile campaigns and at a fixed site from January to December 2023. Screening for HIV, HBV, and HCV was performed using fourth-generation ELISA (Biorad^®^). Data analysis used SPSS with Chi-square test of independence (*p* < 0.05), and multiple logistic regression identified independent risk factors. Among 9646 donors, 87.80% were male (sex ratio 7.19), mostly aged 18–24 (55.93%), with students forming the largest group (58.67%). Mobile units collected 70.80% of donations; 52.60% were repeat donors. Overall TTVI seroprevalence was 9.35%, with HBV (6.29%) most common, followed by HCV (1.78%) and HIV (1.28%). Chi-square tests revealed significant associations between serostatus and donor status, donation site, and occupation, but not sex. Logistic regression identified independent risk factors: age, donor status, and donation site were significantly associated with HIV infection; male sex, older age, occupation, and donor status predicted HBV infection; and only donor status was significantly associated with HCV infection. These findings highlight the need for targeted recruitment and awareness strategies to improve transfusion safety.

## 1. Introduction

Blood transfusion is a vital therapeutic procedure in modern medical practice. It involves the administration of one or more components of blood, such as red blood cells, plasma, platelets, or granulocytes, referred to as labile blood products (LBPs), from one or more healthy individuals (donors) to one or more patients in need (recipients) [1]. Each year, approximately 118.5 million units of blood are collected worldwide [2].

Although transfusion saves many lives, it also carries risks, particularly the transmission of infectious agents including viruses, bacteria, parasites, and prions [3]. Transfusion-transmitted viral infections (TTVIs) represent one of the most serious risks associated with blood transfusion [4]. Among the main pathogens responsible for TTVIs, the human immunodeficiency virus (HIV), hepatitis B virus (HBV), and hepatitis C virus (HCV) are of major concern due to the severity and chronic nature of the diseases they cause [4,5,6].

In Africa, it is estimated that around 500 people acquire a transfusion-transmissible infection (TTI) each day as a result of contaminated blood transfusions [7]. In sub-Saharan Africa, transfused blood is believed to account for 5% to 10% of new HIV infections, and approximately 12.5% of transfused patients are at risk of post-transfusion hepatitis [5]. Several studies in the region have reported high seroprevalence rates of HIV, HBV, and HCV among blood donors [8,9,10]. Worrying prevalence rates have been recorded among donors, including 14.96% in Nigeria and 29.82% in Burkina Faso [11,12]. Assessing TTIs among blood donors is essential to estimate the magnitude of transfusion risk, strengthen prevention strategies, and improve blood safety policies [7,13].

In Benin, data on the prevalence of these infections among blood donors remain scarce. Since 2017, only one published study has investigated TTVIs in Parakou, a municipality in the Borgou department, and it was limited solely to new donors among the blood donor community [14].

Therefore, the present study aims to assess, in 2023, the prevalence and distribution of HIV, HBV, and HCV among all blood donors in the Borgou department of Benin, and to identify potential factors associated with these TTVIs. This study will provide updated data to better inform strategies for blood supply management and to strengthen transfusion safety.

## 2. Materials and Methods

### 2.1. Study Design, Setting, and Study Population

A prospective, cross-sectional study was carried out from 1 January to 31 December 2023, on the serological markers of HIV, HBV, and HCV. The study population consisted of voluntary, non-remunerated blood donors who gave blood at the Borgou Departmental Blood Transfusion Service within the study period. Eligible participants were donors aged between 18 and 64 years, weighed at least 50 kg, and appeared to be in good general health. Donors with contraindications to blood donation and those declared unfit after the pre-donation screening were excluded from the study.

### 2.2. Data Collection

Data were collected using a standardized form based on the pre-donation medical form completed by each donor, as well as records from the Borgou Departmental Blood Transfusion Service. Collected data included socio-demographic characteristics such as age, sex, occupation, donor category (first-time or repeat donor), and donation site (fixed site or mobile unit), along with the results of serological testing for HIV, HBV, and HCV. After obtaining consent, each donor completed the form and had a blood sample drawn in a plain (non-anticoagulated) tube for serological testing.

### 2.3. Laboratory Screening Test

Serological testing for viral markers was performed according to the standard procedures used for blood unit screening in Benin. All donations were tested using fourth-generation enzyme-linked immunosorbent assay (ELISA) kits from Bio-Rad^®^ (Hercules, CA, USA), which detect both antigens and antibodies. The following kits were used:

- HIV: Genscreen ULTRA HIV Ag-Ab—Bio-Rad;
- HBV: Monolisa HBsAg ULTRA—Bio-Rad;
- HCV: Monolisa HCV Ag-Ab ULTRA V2—Bio-Rad.

### 2.4. Data Analysis

Data were entered into Microsoft Excel and used to generate tables and figures. Statistical analyses were performed using SPSS version 20. The Chi-square test of independence was used to assess associations between donor socio-demographic variables and their serological status for HIV, HBV, and HCV. Multiple logistic regression was then performed to identify independent risk factors associated with each infection, after grouping socio-professional categories to avoid overfitting and convergence issues. A *p*-value less than 0.05 was considered statistically significant.

### 2.5. Ethical Consideration

The study proposal was reviewed and approved by the Ethics and Research Committee of the Institute of Applied Biomedical Sciences (CER-ISBA) under number 154 on 22 December 2022. Donors provided verbal consent at the time of donation, which was taken as consent to participate in the study. Donor rights, anonymity, and confidentiality were strictly respected.

## 3. Results

### 3.1. Socio-Demographic Characteristics of the Study Population

A total of 9646 blood donors meeting the inclusion criteria were registered during the study period. Among them, 87.80% were male (95% CI: 87.13–88.44) and 12.20% were female (95% CI: 11.56–12.87), with a male-to-female sex ratio of 7:19. Donors ranged in age from 18 to 65 years. The most represented age group was 18–24 years, accounting for 55.93% of blood donors (95% CI: 54.94–56.92). Students were the most represented occupational category, comprising 58.67% (95% CI: 57.68–59.65) of the sample (Table 1).

The least represented socio-professional group was military and paramilitary personnel (1.93%), followed by medical personnel (2.70%). Most donors were repeat donors (52.60%), while first-time donors accounted for 47.40%. A greater proportion of donations were collected through mobile units (70.80%) compared to a fixed site (29.20%) (Table 1).

### 3.2. Seroprevalence of Transfusion-Transmittable Infections

**Figure 1:**
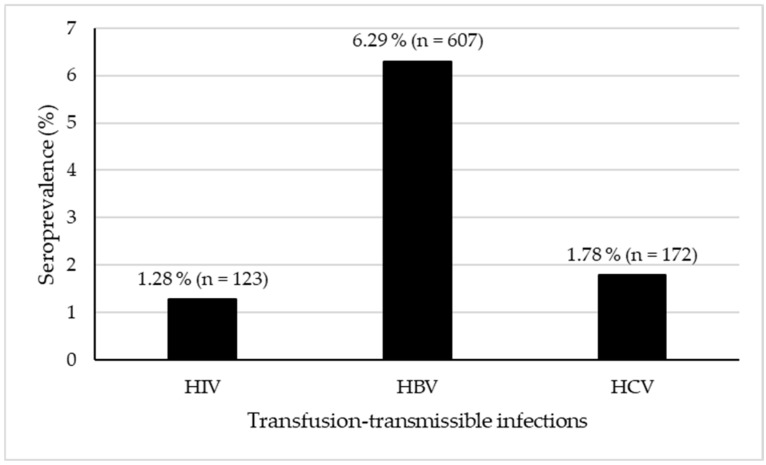
Seroprevalence of HIV, HBV, and HCV among blood donors in Borgou, Benin, in 2023.

The overall seroprevalence of the three transfusion-transmissible infections (HIV, HBV, and HCV) was 9.35% (Figure 1). HBV accounted for the highest proportion at 6.29%, followed by HCV at 1.78%, and HIV at 1.28%.

### 3.3. Seroprevalence of HIV, HBV, and HCV by Donor Characteristics: Distribution and Association Analysis

The seroprevalence of HIV, HBV, and HCV was analyzed according to various socio-demographic characteristics of the blood donors (Table 2).

HIV seroprevalence was 1.31% among male donors and 1.02% among female donors. For HBV, the seroprevalence was 6.41% in males and 5.44% in females, while HCV seroprevalence was 1.78% both in males and females. By age group, HIV seroprevalence ranged from 1.01% (25–30 years) to 1.98% (45–64 years). HBV seroprevalence ranged between 5.41% and 6.47%, and HCV between 0.9% and 2.15% depending on age group. No significant association was observed between sex and the serological status of blood donors for the three viruses in the study population (*p* > 0.05). In contrast, a significant association was observed between age and the serological status of blood donors for HCV only (*p* < 0.05).

First-time donors had higher seroprevalence rates than repeat donors: 1.90% vs. 0.71% for HIV, 10.54% vs. 2.46% for HBV, and 2.6% vs. 1.04% for HCV. A significant association was observed between donor status and the serological status of blood donors for the three viruses in the study population (HIV, HBV, and HCV: *p* < 0.001).

According to socio-professional category, HIV prevalence ranged from 0.38% to 1.55%, HBV from 1.31% to 8.38%, and HCV from 0.77% to 2.16%. The socio-professional category of blood donors was significantly associated with their serological status for HBV (*p* < 0.001) and HCV (*p* = 0.013), but not for HIV (*p* = 0.775).

Finally, seroprevalence at the donation site was higher among donors at mobile collection sites (HIV: 1.51%, HBV: 7.26%, HCV: 1.96%) compared to those at a fixed sites (HIV: 0.71%, HBV: 3.94%, HCV: 1.35%). The donation site was significantly associated with the serological status for HBV (*p* < 0.001), HIV (*p* < 0.001), and HCV (*p* < 0.047) (Table 2).

### 3.4. Transfusion-Transmissible Infections and Associated Risk Factors Among Blood Donors in Borgou

The multivariable logistic regression analyses assessing the associations between demographic and donation-related factors and the seropositivity for HIV, HBV, and HCV among blood donors are presented in Table 3.

Age, donor status, and donation at a mobile site were significantly associated with HIV infection (*p* < 0.05). Specifically, repeat donors had significantly lower odds of HIV infection (AOR: 0.38, 95% CI: 0.25–0.57, *p* < 0.001), while donations collected at mobile sites were associated with higher odds (AOR: 1.77, 95% CI: 1.08–3.02, *p* = 0.029). Age was also a significant predictor, with each additional year of age associated with a 3% increase in the odds of HIV infection (AOR: 1.03, 95% CI: 1.01–1.05, *p* = 0.0042). In contrast, sex and socio-professional category were not significantly associated with HIV status (*p* > 0.05).

For HBV infection, significant predictors included male sex, older age, “Other” socio-professional category, and being a first-time donor. Specifically, male donors had significantly higher odds of HBV infection compared to female donors (AOR: 1.67, 95% CI: 1.28–2.22, *p* < 0.001). Increasing age was also significantly associated, with each additional year corresponding to a 2% increase in the odds of HBV infection (AOR: 1.02, 95% CI: 1.01–1.03, *p* < 0.001). Donors in the “Other” socio-professional category had more than twice the odds of HBV infection (AOR: 2.48, 95% CI: 1.21–6.00, *p* < 0.001). In contrast, repeat donors had substantially lower odds compared to first-time donors (AOR: 0.22, 95% CI: 0.17–0.26, *p* < 0.001).

Regarding HCV infection, only donor status was significantly associated with seropositivity. Specifically, repeat donors were significantly less likely to be HCV positive compared to first-time donors (AOR: 0.42, 95% CI: 0.30–0.59, *p* < 0.001). All other variables, including sex, age, donation site, and occupation, were not significantly associated with HCV infection (*p* > 0.05).

## 4. Discussion

The purpose of this study was to assess the prevalence and distribution of HIV, HBV, and HCV among all blood donors in the Borgou department of Benin in 2023, and to identify potential factors associated with these transfusion-transmissible viral infections (TTVIs). This study revealed distinctive characteristics of blood donors in Borgou in 2023.

In 2023, a total of 9460 blood donations from both male and female donors were collected by the Borgou Departmental Blood Transfusion Service. All donors included in this study (100%) were voluntary, non-remunerated blood donors. This observation contrasts with studies conducted in Mali, which reported 95.47% and 91.40% of donations being family/replacement donations [15,16], as well as with data from a multicountry study conducted in 15 African countries by the Francophone Sub-Saharan Africa Blood Transfusion Research Group, which found that family/replacement donations predominated in most transfusion centers, with voluntary donations accounting for only 15% to 50% [17]. Moreover, this observation aligns with the recommendations of the World Health Organization (WHO), which advocate that all blood donations should come from voluntary, non-remunerated donors to ensure a sufficient, safe, and secure blood supply for the population [18,19]. This observed conformity may be attributed to the many years of awareness campaigns that have helped shift donors’ perceptions, making them understand the importance of voluntary and non-remunerated blood donation. It is also linked to adherence to Benin’s national blood transfusion policy, which prohibits family and replacement donations in accordance with national and international guidelines. The male-to-female sex ratio of 7:19 observed in this study reflects a strong predominance of male donors, who accounted for 87.80% of the study population. The male predominance among blood donors has also been reported in two other studies conducted in Africa [19,20]. This result is consistent with findings from similar studies conducted in the Democratic Republic of Congo (DRC) and Mali [21,22]. This male predominance may be explained by physiological factors and sex-specific contraindications that reduce the frequency of blood donation among women, such as menstruation, pregnancy, and breastfeeding periods. Additionally, sociocultural beliefs still prevalent in many African communities often consider men to be more suitable blood donors, viewing them as stronger, more robust, and in better health than women [15,23]. The 18–24 age group was the most represented, accounting for 55.93%, followed by the 25–30 age group with 20.51%. Therefore, 76.44% of the study population was aged between 18 and 30 years. This predominance of younger donors may be explained by the fact that most blood donors were students (58.67%), who are generally younger. The high representation of this group is also the result of blood donation campaigns that are frequently targeted at schools and universities, where young people are more accessible and more receptive to awareness messages.

The overall seroprevalence observed in this study for the three markers (HIV, HBV, and HCV) was 9.35%. This result is lower than the seroprevalence of 12.6%, reported in a one-year study conducted in Ethiopia in 2019 [7]. However, it is higher than the 1.93% reported in Syria [4], based on a retrospective cross-sectional study that compiled 17 years of screening data. These variations in prevalence rates across studies might be due to differences in the demographic characteristics of donor populations, geographic factors, and variations in diagnostic tools and prescreening procedures. The overall seroprevalence remains below the 10% rejection threshold recommended by Benin’s national blood transfusion guidelines, reflecting the quality of donor selection, a critical factor for transfusion safety. The seroprevalences observed for each of the three markers were 1.28% for HIV, 6.29% for HBV, and 1.78% for HCV, respectively. These values are all lower than those reported by the Blood Transfusion Research Group in Africa, which were 1.84% for HIV, 7.21% for HBV, and 1.99% for HCV [17].

The prevalence of 6.29% for HBV observed in this study is similar to the 6.0% reported in the general population of Benin in a study conducted by Kpossou et al. in 2020 [24]. The prevalence observed in this study is lower than the seroprevalences of 7.9% and 8.01% reported in Mali [25] and the Democratic Republic of Congo (DRC) [19], respectively. However, it is higher than the rates of 4.68% and 5.56% reported by N’dri et al. in Abidjan, Côte d’Ivoire [26], and Bartonjo et al. in Kenya [27], respectively. No significant association was found between the sex of blood donors and their serological status for the three viruses (*p* > 0.05). In contrast, the association between HBV serological status and blood donors’ socio-professional categories was significant. (*p* < 0.001). The lowest prevalences were observed among healthcare workers (0.38%) and military personnel (1.08%). The low seroprevalence among military personnel could be explained by systematic screening followed by mandatory vaccination of seronegative individuals within this profession. Healthcare workers likely adopt preventive measures, including vaccination, due to their awareness of the consequences related to viral hepatitis. The HBV seroprevalence was higher among donors sampled in mobile units (7.26%) compared to those at fixed sites (3.94%), with a statistically significant association between donation site and HBV serological status (*p* < 0.001). The lower HBV seroprevalence observed among donors at fixed sites may be attributed to the higher proportion of repeat donors who regularly donate at these locations. Repeat donors are generally considered a lower-risk group due to prior screening and greater awareness of eligibility and transfusion safety criteria. In contrast, mobile collection units tend to attract a larger proportion of first-time donors, who may be less familiar with screening requirements and may originate from more diverse or less-controlled environments, potentially increasing the likelihood of HBV exposure in this subgroup. Among first-time donors, the HBV seroprevalence was 10.54%, slightly lower than the 12.74% reported by Attinsounon et al. [14] in 2017 in Parakou municipality in Benin.

The HBV seroprevalence of 10.54% among new donors was significantly higher than the 2.46% observed among repeat donors, with a statistically significant difference (*p* < 0.001). This difference is likely due to the healthier lifestyle of repeat donors, who have been educated about the precautions and behaviors necessary to prevent transmissible diseases, given their status as regular donors. Consequently, blood donations from repeat donors are generally safer than those from new donors.

The HCV seroprevalence of 1.78% observed in this study is lower than the rates of 3.6%, 3.71%, and 8.69% reported in Nigeria [11], Equatorial Guinea [28], and Burkina Faso [14], respectively. However, it is higher than the rates of 0.20%, 0.50%, 1.3%, and 1.5%, found in Mali [25], Cameroon [13,29], and Mali [30], respectively.

A statistically significant association was found between donors’ status and their HCV serological status, with a prevalence of 2.6% among new donors compared to 1.04% among repeat donors (*p* < 0.001).

The student group showed the highest HCV prevalence (1.16%), followed by the military and paramilitary group (1.15%). The lowest prevalence was recorded among healthcare workers (0.77%), suggesting either lower exposure or more rigorous adherence to infection prevention measures in this group. A statistically significant association was found between professional categories and HCV infection (*p* = 0.015).

The HIV seroprevalence in this study was 1.28%. It is lower than the rates of 5.30% and 7.83% reported in Mali [16] and Equatorial Guinea [28], respectively. However, it is higher than the rates of 0.15‰ reported in a study conducted in Morocco [31]. These variations in prevalence rates across studies are likely attributable to differences in local risk behaviors and demographic characteristics of donor populations. Additionally, disparities in study design, occupational and geographic factors, as well as differences in diagnostic tools and prescreening procedures, may further contribute to the observed discrepancies. For HIV infection, no significant association was found with sex or socio-professional categories (*p* > 0.05), suggesting that these variables did not influence HIV seropositivity in the study population. However, a significant association was observed with donor status and donation site (*p* < 0.05). This finding underscores the importance of reinforcing pre-donation screening and education, particularly for first-time donors, who may not be as familiar with transfusion safety criteria or may represent a higher-risk group.

## 5. Conclusions

This study, conducted on 9646 donations exclusively from voluntary non-remunerated blood donors at the Borgou Departmental Blood Transfusion Service, revealed an overall seroprevalence of 9.35% for the three viruses (HIV, HBV, and HCV), with variations between each virus. The highest prevalence was observed for HBV (6.29%), followed by HCV (1.78%), and HIV (1.28%). The serological status of blood donors for the three viruses was significantly associated with donor status (first-time vs. repeat). Additionally, significant associations were observed between socio-professional category and serological status for HBV and HCV, as well as between donation site and serological status for the three viruses, but not with sex. These findings highlight the importance of focusing on first-time donors and mobile collection sites to improve transfusion safety. It is therefore essential to tailor blood donation promotion and donor recruitment strategies by considering these variations in order to enhance transfusion safety.

## Figures and Tables

**Table 1:** Socio-demographic characteristics of blood donors in Borgou, Benin, in 2023 (*n* = 9646).

Characteristic	Frequency (*n*)	Percentage (%)	95% Confidence Interval (CI)
**Sex**			
Male	8469	87.80	[87.13–88.44]
Female	1177	12.20	[11.56–12.87]
**Age group (years)**			
18–24	5395	55.93	[54.94–56.92]
25–30	1978	20.51	[19.71–21.32]
31–44	1718	17.81	[17.06–18.59]
45–64	555	5.75	[5.31–6.24]
**Donor status**			
First-time donor	4572	47.40	[46.40–48.40]
Repeat donor	5074	52.60	[51.60–53.60]
**Socio-professional category**			
Student	5659	58.67	[57.68–59.65]
Salaried worker	1410	14.62	[13.93–15.34]
Artisan	973	10.09	[9.50–10.70]
Medical/paramedical staff	260	2.70	[2.39–3.04]
Military/paramilitary	186	1.93	[1.67–2.22]
Other	1158	12.00	[11.37–12.67]
**Donation site**			
Mobile unit	6829	70.80	[69.88–71.70]
Fixed site	2817	29.20	[28.30–30.12]

**Table 2:** Seroprevalence of HIV, HBV, and HCV by socio-demographic characteristics of blood donors in Borgou, Benin (*n* = 9646).

Characteristic	Total Number	HIV *n* (%)	HBV *n* (%)	HCV *n* (%)
**Sex**				
Male	8469	111 (1.31%)	543 (6.41%)	151 (1.78%)
Female	1177	12 (1.02%)	64 (5.44%)	21 (1.78%)
*p*-value		>0.05	>0.05	>0.05
**Age group (years)**				
18–24	5395	68 (1.26%)	349 (6.47%)	116 (2.15%)
25–30	1978	20 (1.01%)	126 (6.37%)	31 (1.57%)
31–44	1718	24 (1.4%)	102 (5.94%)	20 (1.16%)
45–64	555	11 (1.98%)	30 (5.41%)	5 (0.9%)
*p*-value		>0.05	>0.05	**<0.05**
**Donor status**				
First-time donor	4572	87 (1.9%)	482 (10.54%)	119 (2.6%)
Repeat donor	5074	36 (0.71%)	125 (2.46%)	53 (1.04%)
*p*-value		**<0.001**	**<0.001**	**<0.001**
**Socio-professional category**				
Student	5659	71 (1.25%)	374 (6.61%)	122 (2.16%)
Salaried worker	1410	17 (1.21%)	54 (3.83%)	13 (0.92%)
Artisan	973	14 (1.44%)	72 (7.4%)	14 (1.44%)
Medical/paramedical staff	260	1 (0.38%)	7 (2.69%)	2 (0.77%)
Military/paramilitary	186	2 (1.08%)	3 (1.61%)	4 (2.15%)
Other	1158	18 (1.55%)	97 (8.38%)	17 (1.47%)
*p*-value		0.775	**<0.001**	**0.013**
**Donation site**				
Mobile unit	6828	103 (1.51%)	496 (7.26%)	134 (1.96%)
Fixed site	2818	20 (0.71%)	111 (3.94%)	38 (1.35%)
*p*-value		**<0.001**	**<0.001**	**0.047**

*n*: number of positive cases for each infection; (%) percentage of positive cases for each infection; *p*-values indicate the results of Chi-square test of independence assessing the association between each characteristic and the serological status of blood donors for each virus. A *p*-value < 0.05 was considered statistically significant.

**Table 3:** Multiple logistic regression results for risk factors associated with transfusion-transmissible infections (HIV, HBV, and HCV) among blood donors in Borgou (2023).

		HIV		HBV		HCV	
Variable	Category	AOR [95% CI]	*p*-Value	AOR [95% CI]	*p*-Value	AOR [95% CI]	*p*-Value
Sex	Female	–	–	–	–	–	–
	Male	1.65 [0.94–3.19]	0.104	1.67 [1.28–2.22]	***p*** ** < 0.001**	1.19 [0.76–1.95]	0.470
Age (continuous)	Per year increase	1.03 [1.01–1.05]	**0.0042**	1.02 [1.01–1.03]	***p*** ** < 0.001**	0.99 [0.96–1.01]	0.289
Socio-professional category	Medical/paramedical staff	–	–	–	–	–	–
	Other	3.22 [0.66–58.3]	0.257	2.48 [1.21–6.00]	***p*** ** < 0.001**	1.72 [0.49–10.9]	0.470
	Salaried/Military	2.83 [0.58–51.2]	0.313	1.16 [0.56–2.85]	0.710	1.39 [0.39–8.84]	0.662
	Student/Artisan	3.30 [0.72–58.6]	0.239	2.12 [1.06–5.04]	0.0557	2.03 [0.63–12.4]	0.327
Donor status	First-time donor	–	–	–	–	–	–
	Repeat donor	0.38 [0.25–0.57]	***p*** ** < 0.001**	0.22 [0.17–0.26]	***p*** ** < 0.001**	0.42 [0.30–0.59]	***p*** ** < 0.001**
Donation site	Fixed site	–	–	–	–	–	–
	Mobile site	1.77 [1.08–3.02]	**0.0286**	1.20 [0.96–1.51]	0.114	0.98 [0.68–1.47]	0.935

## Data Availability

All data generated and/or analyzed during the current study are included in this published article.

## Abbreviations

The following abbreviations are used in this manuscript:

LBPs	labile blood products
TTVIs	transfusion-transmitted viral infections
HIV	human immunodeficiency virus
HBV	hepatitis B virus
HCV	hepatitis C virus
SPSS	Statistical Package for the Social Sciences
HBsAg	hepatitis B surface antigen
HCV Ag-Ab	hepatitis C virus antigen-antibody combination
ELISA	enzyme-linked immunosorbent assay
